# Supplementary material for: MET mutation causes muscular dysplasia and arthrogryposis
Source: EMBO Mol Med. 2019 Feb 18;11(3):e9709. doi: 10.15252/emmm.201809709 (PMC6404111; doi:10.15252/emmm.201809709)
Supplement: Supplementary file 1 — Appendix [file EMMM-11-e9709-s001.pdf]

Table of Contents

1) **Appendix Table S1**
2) **Appendix Table S2**
3) **Appendix Statistical Analysis**

Appendix Table S1: Depth and coverage of whole-exome sequencing

Sample	**II:14**	**III:14**	**III:16**	**III:22**	**IV:8**
Total:	100%	100%	100%	100%	100%
Duplicate:	9.29%	9.15%	10.72%	9.30%	9.63%
Mapped:	99.96%	99.95%	99.46%	99.94%	99.96%
Total effective yield(Mb):	14034.29	12014.79	13977.26	13195.62	14360.81
Effective yield on target(Mb):	8807.90	7526.78	8846.36	8170.70	8968.10
Fraction of effective bases on target:	62.8%	62.6%	63.3%	61.9%	62.4%
Fraction of effective bases on or near target:	87.0%	87.4%	86.7%	86.8%	87.0%
Average sequencing depth on target:	174.79	149.37	175.56	162.15	177.97
Bases covered on target:	50294918	50287105	50288560	50289891	50337216
Coverage of target region:	99.8%	99.8%	99.8%	99.8%	99.9%
Fraction of target covered with at least 100x:	73.4%	68.8%	76.1%	73.1%	73.6%
Fraction of target covered with at least 50x:	93.5%	93.0%	95.0%	94.3%	93.7%
Fraction of target covered with at least 20x:	98.8%	98.8%	99.3%	99.0%	98.9%
Fraction of target covered with at least 10x:	99.4%	99.5%	99.6%	99.5%	99.6%
Fraction of target covered with at least 4x:	99.7%	99.7%	99.8%	99.7%	99.8%

Appendix Table S2: Detailed information for *MET* c.A3701G after annotation

cDNA	*MET* c.A3701G
Amino acid change	*MET* p.Tyr1234Cys
In dbSNP or not	NONE
Function GVS	Missense mutation
ConsScore GERP	5.46
PhastCons	1
PolyPhen 2	1/1(Probably Damaging)
Mutation Taster	194/225(Disease-causing)

Appendix Statistical Analysis

**Figure 2**			
**2B body weight**			
	Significant	Summary	Individual *P* Value
WT vs. Hetero	YES	*	0.04579
**2H Number of Myofibers**			
	Significant	Summary	Individual *P* Value
Paraspinal WT vs. Hetero	YES	*	0.01549
Forelimb WT vs. Hetero	YES	**	0.00001
Hindlimb WT vs. Hetero	YES	*	0.02300
Hand WT vs. Hetero	YES	**	0.00002
Foot WT vs. Hetero	No	NS	0.22568

**Figure 3**			
**3G Positive rate of Ki67**			
	Significant	Summary	Individual *P* Value
WT vs. Hetero	YES	**	0.00005
WT vs. Homo	YES	**	0.00001
Hetero vs. Homo	YES	**	0.00017

**Figure EV4**			
**4A Weight of E14.5**			
	Significant	Summary	Individual *P* Value
WT vs. Hetero	NO	NS	0.50900
WT vs. Homo	YES	*	0.02000
Hetero vs. Homo	YES	*	0.04800
**4E Positive rate of Ki67**			
	Significant	Summary	Individual *P* Value
WT vs. Hetero vs. Homo	NO	NS	0.69400

**4F Positive rate of TUNEL**			
	Significant	Summary	Individual *P* Value
WT vs. Hetero vs. Homo	NO	NS	0.77400

**Figure EV5**			
**5A Weight of E16.5**			
	Significant	Summary	Individual *P* Value
WT vs. Hetero	NO	NS	0.72233
WT vs. Homo	YES	*	0.03234
Hetero vs. Homo	YES	*	0.02465
**5B Number of Myofibers**			
	Significant	Summary	Individual *P* Value
Forelimb WT vs. Hetero	YES	**	0.00009
Hindlimb WT vs. Hetero	YES	*	0.04300
Spine WT vs. Hetero	YES	*	0.02300
Spine WT vs. Homo	YES	**	0.00034
Spine Hetero vs. Homo	YES	**	0.00036
**5D Positive rate of TUNEL**			
	Significant	Summary	Individual *P* Value
WT vs. Hetero vs. Homo	NO	NS	0.41800
