# Supplementary material for: MET mutation causes muscular dysplasia and arthrogryposis
Source: EMBO Mol Med. 2019 Feb 18;11(3):e9709. doi: 10.15252/emmm.201809709 (PMC6404111; doi:10.15252/emmm.201809709)
Supplement: Supplementary file 2 — Expanded View Figures PDF [file EMMM-11-e9709-s002.pdf]

Expanded View Figures

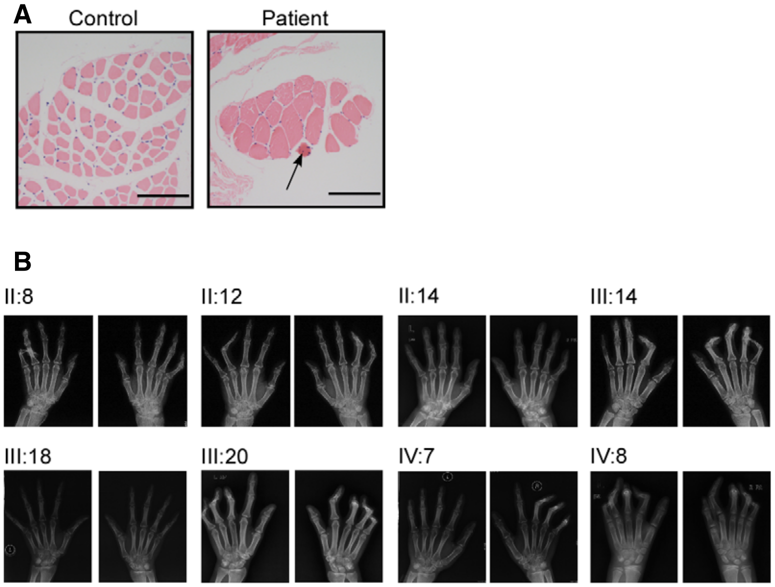

Figure EV1. Histological study and X-ray scan of arthrogryposis patients.

A HE staining of lumbrical samples from patient IV:8 and the age-/gender-matched control was conducted. Centrally located muscles were indicated by a black arrow. Scale bars, 100 μm.

B X-ray scan of the patients' hands was conducted.

 © 2019 The Authors

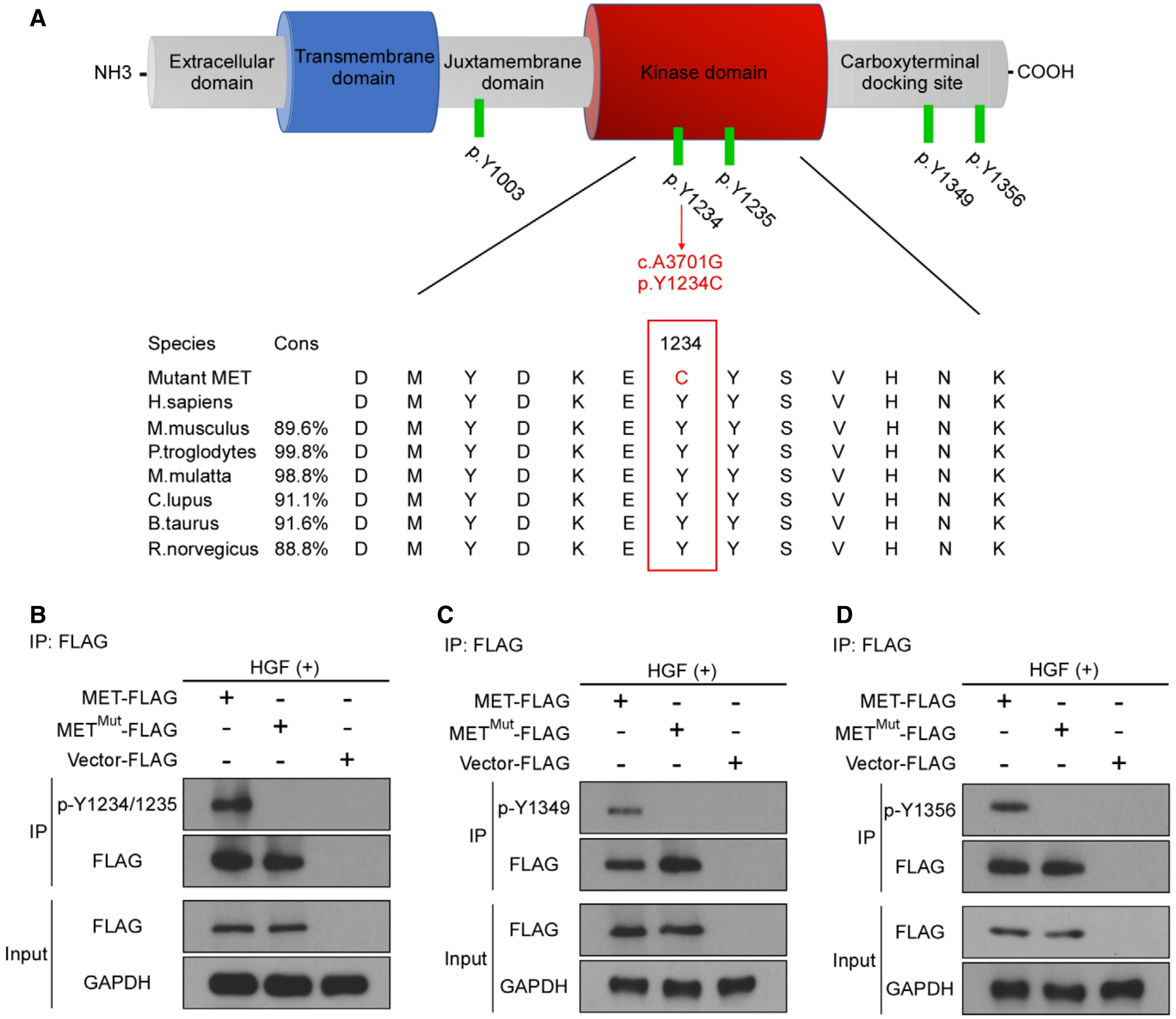

Figure EV2. *MET* p.Y1234C mutation caused the dysfunction of the phosphorylation of MET protein.

A Upper panel shows the protein structure of MET. Green bars represent tyrosine phosphorylation sites, and red arrow indicates the mutation that was observed in our arthrogryposis pedigree. Lower panel shows phylogenetic conservation of mutated residues and homology among different species of *MET* gene, and cons means conservation.

B–D 293T cells were transfected with pCMV-C-FLAG-MET, pCMV-C-FLAG-MET^{Mut}, or pCMV-C-FLAG-Vector, and 48 h post-transfection, cells were treated with 10 ng/ml recombinant human HGF for 1 h. Immunoprecipitation was carried out with anti-FLAG antibody and followed by immunoblotting with anti-FLAG antibody and anti-p-MET$^{Y1234/1235}$ (B), anti-p-MET^{Y1349} (C), and anti-p-MET^{Y1356} (D) antibodies. Western blot pictures representative of $n = 3$ experiments.

Source data are available online for this figure.

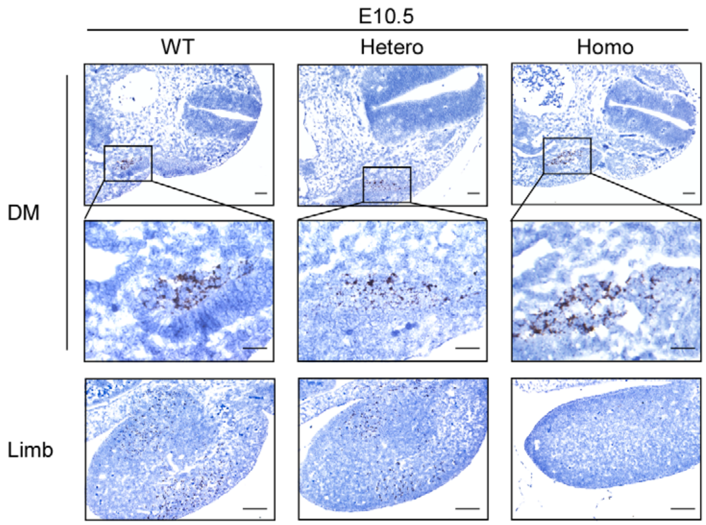

Figure EV3. *In situ* hybridization of E10.5 embryos using *Met* probe.

Met expression (brown signal) was detected through *in situ* hybridization in dermomyotome (DM) and limb bud of E10.5 embryos from all three genotypes, $n = 3$. Scale bars, 200 μm. WT means wild types, Hetero means heterozygotes, and Homo means homozygotes.

© 2019 The Authors

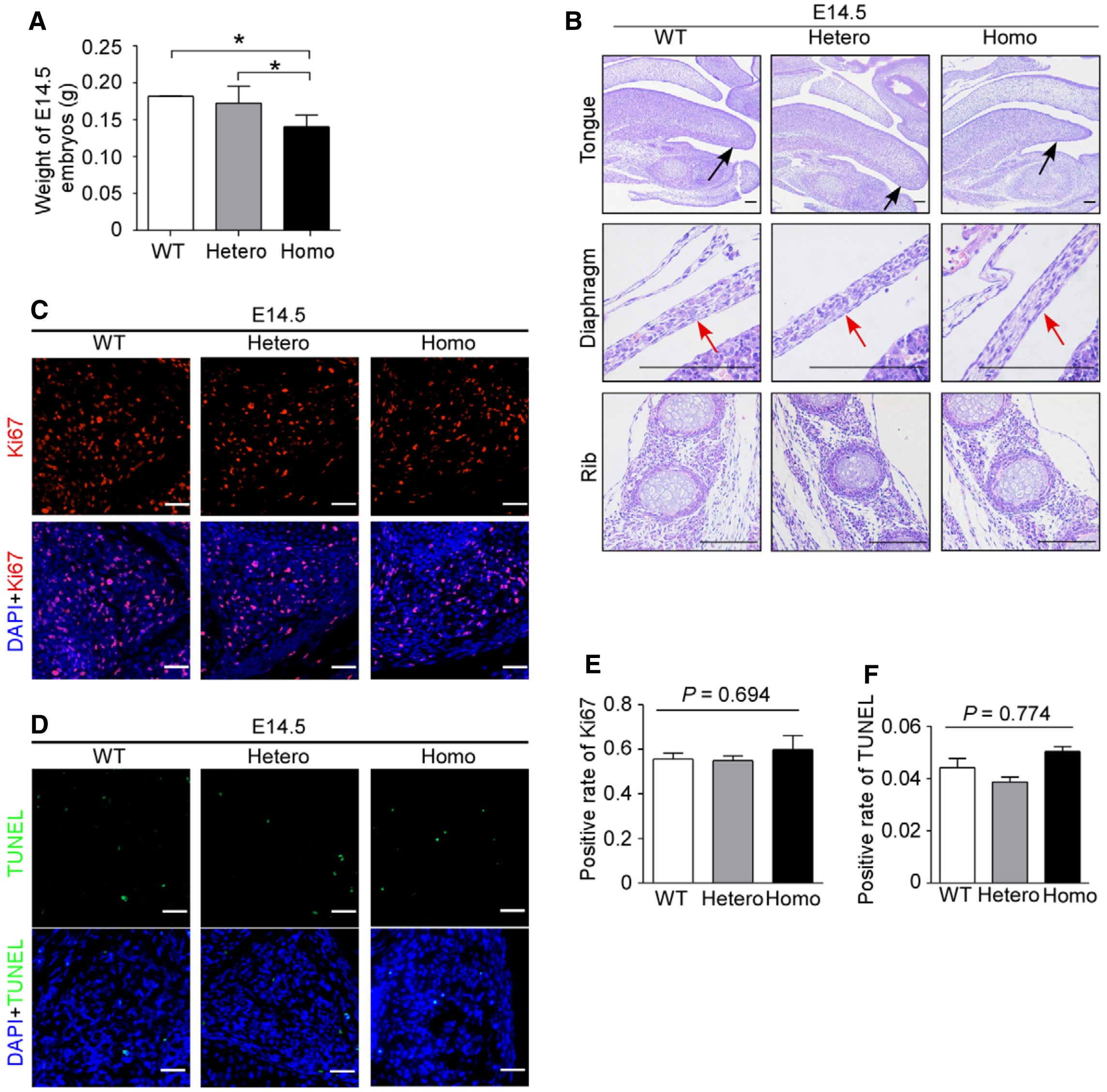

Figure EV4. *Met* mutation had no effect on the formation of primary muscle fiber.

A Graph indicates the weight of E14.5 embryos of three genotypes. Bars show mean ± SD. Sample size: WT (n = 5), Hetero (n = 5), and Homo (n = 5). *$P < 0.05$, by one-way ANOVA and followed by Dunnett's *post hoc* test.

B HE staining of front tongue, diaphragm, and rib from E14.5 embryos was performed. Front tongue was denoted by black arrow, and diaphragm was indicated by a red arrow. Scale bars, 100 μm.

C Anti-Ki67 antibody was used to label proliferative myoblasts (red fluorescence) with DAPI-labeled nuclei (blue fluorescence) in paraspinal muscles of E14.5 embryos. Scale bars, 25 μm.

D TUNEL assay was conducted to reveal apoptotic myoblasts (green fluorescence) with DAPI-labeled nuclei (blue fluorescence) in paraspinal muscles of E14.5 embryos. Scale bars, 25 μm.

E Positive rate of Ki67-labeled nuclei in (C) was quantified. Bars show mean ± SD.

F Positive rate of TUNEL-labeled nuclei in (D) was quantified. Bars show mean ± SD.

Data information: In (E) and (F), n = 3 with more than 150 cells analyzed per n, by chi-square test (χ^2 test). WT means wild types, Hetero means heterozygotes, and Homo means homozygotes.

© 2019 The Authors

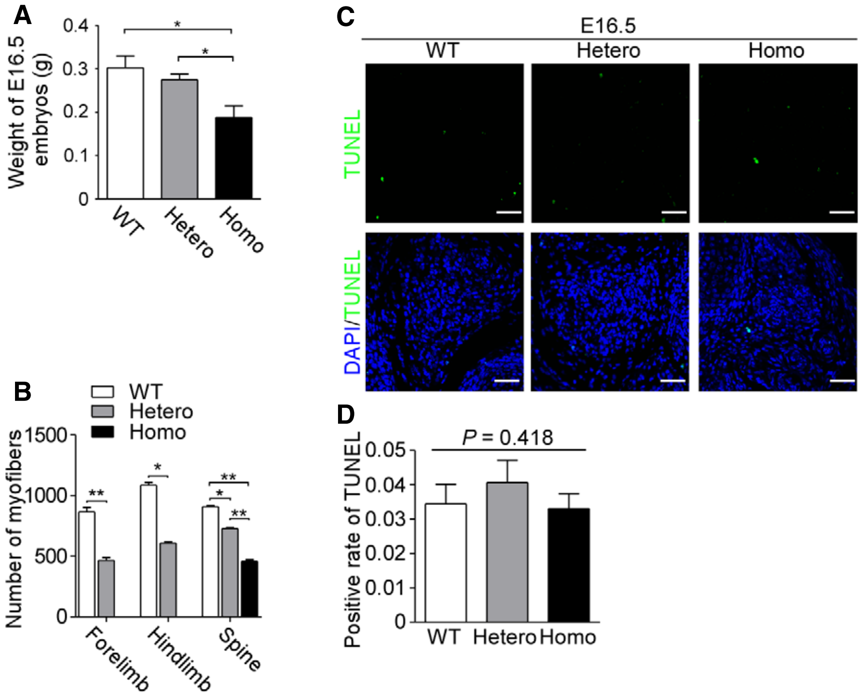

Figure EV5. ***Met* mutation suppressed the proliferation of secondary myoblasts.**

A Graph indicates the weight of E16.5 embryos of three genotypes. Bars show mean ± SD. Sample size: WT (n = 4), Hetero (n = 4), and Homo (n = 4). $^*P < 0.05$, by one-way ANOVA and followed by Dunnett's *post hoc* test.

B The myofiber numbers of forelimb, hindlimb, and paraspinal muscles of E16.5 embryos were qualified. Bars show mean ± SD. n = 3 with more than 400 myofibers analyzed per n. $^*P < 0.05$, $^{**}P < 0.01$, by one-way ANOVA followed by Dunnett's *post hoc* test.

C TUNEL assay was conducted to indicate apoptotic myoblasts (green fluorescence) with DAPI-labeled nuclei (blue fluorescence) in paraspinal muscle of E16.5 embryos, n = 3. Scale bars, 25 μm.

D Bar graph showing statistical analysis of positive rate of TUNEL-labeled nuclei in (C). Bars show mean ± SD.

Data information: In (D), n = 3 with more than 150 cells analyzed per n, by chi-square test (χ^2 test). WT means wild types, Hetero means heterozygotes, and Homo means homozygotes.

 © 2019 The Authors
