# Supplementary material for: MET mutation causes muscular dysplasia and arthrogryposis
Source: EMBO Mol Med. 2019 Feb 18;11(3):e9709. doi: 10.15252/emmm.201809709 (PMC6404111; doi:10.15252/emmm.201809709)
Supplement: Supplementary file 3 — Table EV1 [file EMMM-11-e9709-s003.docx]

**Table Expanded View 1: Clinical features of individuals with *MET* mutation.**

| Characteristics | I:2 | II:2 | II:8 | II:10 | II:12 | II:14 | III:14 | III:16 | III:18 | III:20 | IV:7 | IV:8 |
| --- | --- | --- | --- | --- | --- | --- | --- | --- | --- | --- | --- | --- |
| Camptodactyly | + | + | + | + | + | + | + | + | + | + | + | + |
| Absent flexion crease | UN | + | + | + | + | + | + | + | - | + | + | + |
| Overriding finger | - | - | - | - | - | - | - | - | - | - | - | - |
| Limited forearm supination | UN | - | + | - | + | + | + | - | - | + | + | + |
| Calcaneovalgus | - | - | - | - | - | - | - | - | - | - | - | - |
| Verticle talus | - | - | - | - | - | - | - | - | - | - | - | - |
| Metatarsus varus | - | - | - | - | - | - | - | - | - | - | - | - |
| Club foot | - | - | - | - | - | - | - | - | - | - | - | - |

This table provides a summary of clinical features from affected individuals in arthrogryposis pedigree. +, presence of finding; -, absence of finding; UN, abbreviation for unknown.
