# Supplementary material for: MET mutation causes muscular dysplasia and arthrogryposis
Source: EMBO Mol Med. 2019 Feb 18;11(3):e9709. doi: 10.15252/emmm.201809709 (PMC6404111; doi:10.15252/emmm.201809709)

Figure EV2D

1. IP-Anti-p-Y1356

Order of samples: MET, MET^{Mut}, and Vector

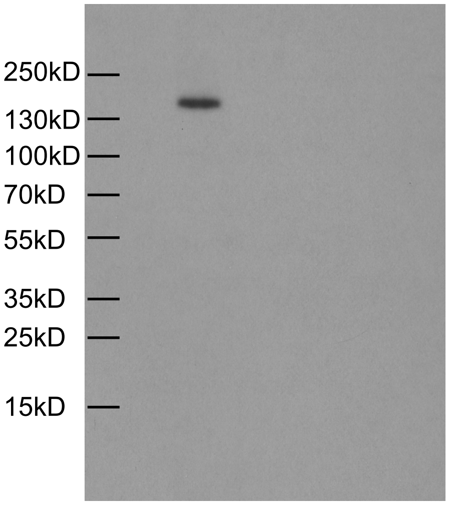

2. IP-Anti-FLAG

Same order as left

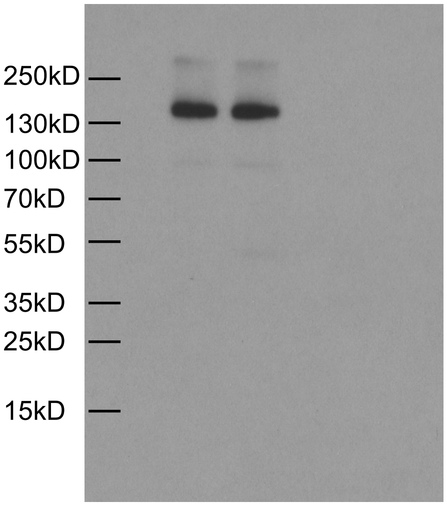

3. Input-Anti-FLAG

Same order as above

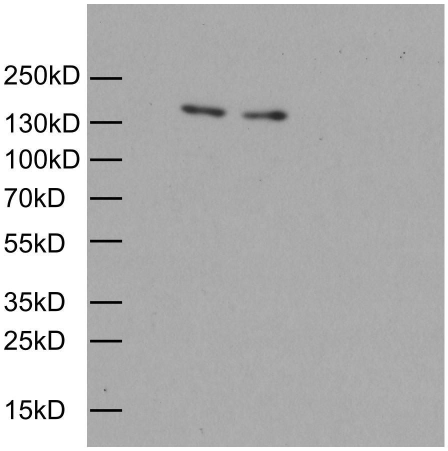

4. Input-Anti-GAPDH

Same order as above

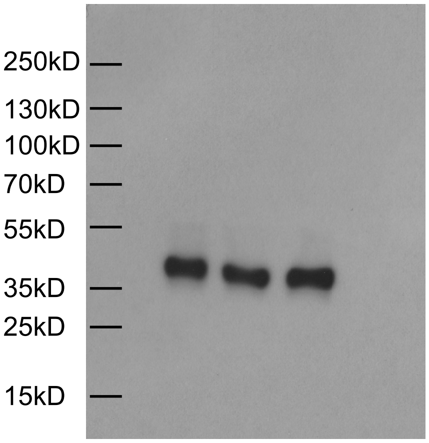

Supplement: Supplementary file 4 — Source Data for Expanded View [file EMMM-11-e9709-s005.zip › souce_data_for_fig_EV2D.pdf]
