# Supplementary material for: MET mutation causes muscular dysplasia and arthrogryposis
Source: EMBO Mol Med. 2019 Feb 18;11(3):e9709. doi: 10.15252/emmm.201809709 (PMC6404111; doi:10.15252/emmm.201809709)

Figure 1J

1. Tyrosine kinase activity

Order of samples: EGFR, NC, Vector, MET, and MET^{Mut}

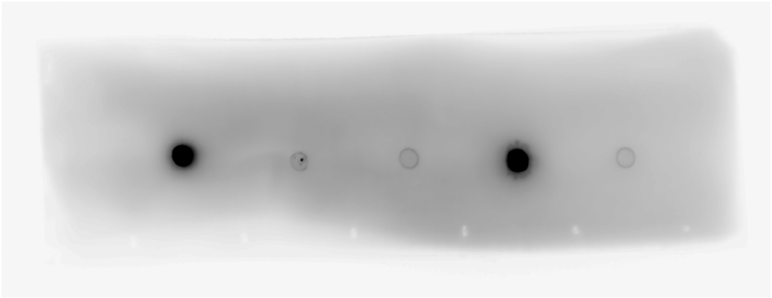

2. Anti-FLAG

Order of samples: MET, MET^{Mut}, and Vector

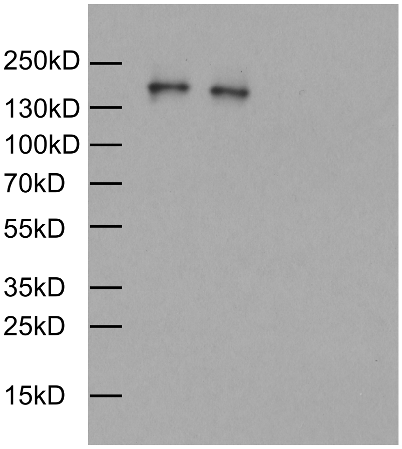

3. Anti-GAPDH

Order of samples: MET, MET^{Mut}, and Vector

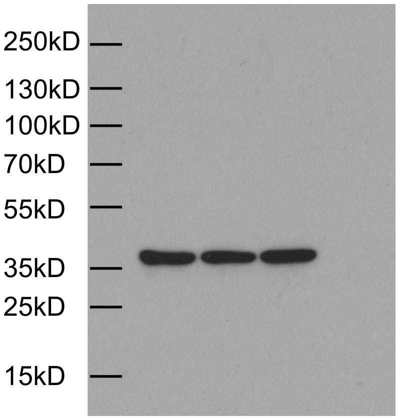

Supplement: Supplementary file 6 — Source Data for Figure 1J [file EMMM-11-e9709-s004.pdf]
